# Leishmanicidal Activity and Ultrastructural Changes of Maslinic Acid Isolated from *Hyptidendron canum*

**DOI:** 10.1155/2021/9970983

**Published:** 2021-05-31

**Authors:** Jéssica Adriana Jesus, Márcia Dalastra Laurenti, Matheus Lopes Silva, João Henrique Ghilardi Lago, Luiz Felipe Domingues Passero

**Affiliations:** ^1^Laboratory of Pathology of Infectious Diseases (LIM50), Department of Pathology, Medical School of São Paulo University, Av. Dr. Arnaldo, 455. Cerqueira César, SP 01246-903, Brazil; ^2^Centre of Natural and Human Sciences, Federal University of ABC (UFABC), Santo André 09210-580, Brazil; ^3^São Paulo State University (UNESP), Institute of Biosciences, São Vicente. Praça Infante Dom Henrique, s/n, São Vicente, SP 11330-900, Brazil; ^4^São Paulo State University (UNESP), Institute for Advanced Studies of Ocean, São Vicente. João Francisco Bensdorp, 1178, São Vicente, SP 11350-011, Brazil

## Abstract

The therapeutic arsenal for the treatment of leishmaniasis is limited and has serious obstacles, such as variable activity, high toxicity, and costs. To overcome such limitations, it becomes urgent to characterize new bioactive molecules. Plants produce and accumulate different classes of bioactive compounds, and these molecules can be studied as a strategy to combat leishmaniasis. The study presented herein evaluated the leishmanicidal effect of maslinic acid isolated from the leaves of *Hyptidendron canum* (Lamiaceae) and investigated the morphological that occurred on *Leishmania (Leishmania) infantum* upon treatment. Maslinic acid was active and selective against promastigote and amastigote forms in a dose-dependent manner. Additionally, it was not toxic to peritoneal macrophages isolated from golden hamsters, while miltefosine and amphotericin B showed mild toxicity for macrophages. Morphological changes in promastigotes of *L. (L.) infantum* treated with maslinic acid were related to cytoplasmic degeneration, intense exocytic activity, and blebbing in the kDNA; disruption of mitochondrial cristae was observed in some parasites. The nucleus of promastigote forms seems to be degraded and the chromatin fragmented, suggesting that maslinic acid triggers programmed cell death. These results indicate that maslinic acid may be an interesting molecule to develop new classes of drugs against leishmaniasis.

## 1. Introduction

Leishmaniasis is an important neglected tropical disease caused by different species of the protozoan parasite that belong to the genus *Leishmania*. It affects vulnerable people in approximately 100 countries, with 12 million cases detected worldwide per year. Additionally, one billion people live in areas at risk of transmission [1]. The treatment of leishmaniasis relies on the use of the first-line drug pentavalent antimonial that is highly toxic for humans, limiting the use and adherence of patients with leishmaniasis [2]. Second-line drugs, such as amphotericin B, miltefosine, or paromomycin, can be used in patients that do not tolerate treatment with antimonials, or in cases that it fails at eliminating parasites [3]. These drugs show different degrees of effectiveness in leishmaniasis, which depends on the infecting species and the clinical form of leishmaniasis, and similarly to antimonials, second-line drugs induce side effects that limit their use [4, 5]. As a consequence of the small number of available drugs currently used in therapy as well as different levels of therapeutic activity, it becomes urgent to implement leishmanial chemotherapy with new strategies. In this regard, it has been demonstrated that medicinal plants can be considered interesting scaffolds to isolate and characterize highly active molecules to be used in leishmaniasis [6–8].

*Hyptidendron canum* (syn. *Hyptis cana*) belongs to Lamiaceae and it is native to the Brazilian “cerrado” region (savannah like biome), especially in Goias, Mato Grosso, and Minas Gerais States [9]. Infusions and decoctions produced with the leaves and roots of *H. canum* have been used in folk medicine to treat inflammatory processes and infections caused by parasites and viruses [10–12]. All these pharmacological activities can be associated with the presence of active secondary metabolites that have been obtained from different organs of *H. canum* [13], such as the pentacyclic triterpenes betulinic, ursolic, and maslinic acids [14].

In leishmaniasis treatment, the most studied triterpene so far is ursolic acid, including *in vitro* and *in vivo* assays. The first report showed that this metabolite was active against promastigote and amastigote stages of *L. (L.) major* and *L. (L.) donovani* [15]; further studies showed that ursolic acid triggered programmed cell death in *L. (L.) amazonensis* [16]. Besides, golden hamsters with visceral leishmaniasis and BALB/c mice with cutaneous leishmaniasis showed a significant reduction in tissue parasitism during the treatment with this triterpene [16, 17], and such activity was reported to be linked with the immunomodulatory activity [7, 17]. Additionally, in experimental therapeutic approaches using combinatorial therapy, it was shown that ursolic acid potentialized the activity of glucantime in cutaneous leishmaniasis [18]. Although plenty of studies have shown the bioactivity of ursolic acid against leishmaniasis, few studies were performed to evaluate the antileishmanial activity of other related pentacyclic triterpenes, produced and accumulated by *H. canum*, as is the case of maslinic acid.

Maslinic acid is widely distributed among species, including edible plants such as spinach, eggplant, olive, and basil [19–23], suggesting that this triterpene has no toxicity for humans. In the present work, it has been demonstrated, for the first time, that maslinic acid isolated from the leaves of *H. canum* inhibited the growth of both stages of *L. (L.) infantum* and parasite death was associated with leishmanial organelle disorganization.

## 2. Materials and Methods

### 2.1. General

Column and thin layer chromatographic procedures were performed, respectively, using silica gel 60 (230 − 400 mesh, Merck) and silica gel 60 PF254 (Merck). Semipreparative HPLC procedure was performed on a Dionex Ultimate 3000 chromatography using a Luna Phenomenex C_18_ column (10 × 250 mm particle and pore size of 5 *μ*m and 175 Å, respectively), and detector UVD-DAD, 170 V. NMR spectra were recorded on a Bruker Ultrashield 300 Avance III spectrometer (Billerica, MA, USA) operating at 300 and 75 MHz with ^1^H and ^13^C nuclei, respectively. All samples were dissolved in CDCl_3_ and drops of CD_3_OD (both solvents from Sigma-Aldrich) were added to make a homogeneous medium. ESI-HRMS spectra were measured using an ESI ion source in negative mode on a Bruker Daltonics MicroTOF QII (Billerica, MA, USA).

### 2.2. Plant Material and Preparation of Crude Extract

Fresh leaves of *Hyptidendron canum* (Pohl ex Benth.) Harley (Lamiaceae) were collected in Goiania, GO, Brazil, in December 2019. The plant material was compared with a voucher specimen SP205332 deposited in the Herbarium of *Instituto de Botânica de São Paulo*, SP, Brazil. This study was approved by Ministério do Meio Ambiente, Brazil (SisGen N. A095CE9). The leaves were dried at 35°C for 72 h, milled, and the obtained material (312 g) was exhaustively extracted using MeOH at room temperature, to afford 11 g of MeOH extract after evaporation of the solvent under reduced pressure.

### 2.3. Isolation of Maslinic Acid

Part of MeOH extract from leaves of *H. canum* (8 g) was resuspended in MeOH : H_2_O_2_ : 1 and sequentially partitioned with hexane and EtOAc to obtain, respectively, 3.4 g and 2.7 g of each organic phase. Part of the EtOAc phase (2.5 g) was subjected to column chromatography under silica gel eluted with increasing amounts of EtOAc in hexane to give six fractions (A–F). Fraction C (154 mg) was subjected to column chromatography over silica gel eluted with hexane:EtOAc (8 : 2, 7 : 3, and 1 : 1) to give a mixture of triterpenes on fraction C-3 (34 mg). This subfraction was purified by semipreparative HPLC using MeOH : H_2_O 9 : 1 as a mobile phase to afford pure maslinic acid (17.6 mg).

#### 2.3.1. Maslinic Acid

^1^H NMR (CDCl_3_ + drops of CD_3_OD, 300 MHz): *δ* 5.32 (*t*, *J* = 3.7 Hz, H-12), 3.71 (m, H-2), 3.01 (d, *J* = 9.5 Hz), 1.15 (s, H-27), 1.03 (s, H-23), 1.00 (s, H-25), 0.93 (s, H-30), 0.91 (s, H-29), 0.84 (s, H-24), 0.78 (s, H-26). ^13^C NMR (CDCl_3_ + drops of CD_3_OD, 75 MHz): *δ* 178.5 (C-28), 144.7 (C-13), 122.0 (C-12), 83.4 (C-3), 68.5 (C-2), 55.2 (C-5), 47.5 (C-1 and C-9), 46.3 (C-17), 46.1 (C-19), 41.6 (C-14), 41.1 (C-18), 39.2 (C-4), 39.1 (C-8), 38.1 (C-10), 33.9 (C-29), 33.8 (C-21), 32.9 (C-7 and C-22), 30.6 (C-20), 28.5 (C-23), 27.5 (C-15), 25.8 (C-27), 23.4 (C-11 and C-16), 23.3 (C-30), 18.3 (C-6), 17.4 (C-24), 17.1 (C-26), 16.4 (C-25). ESI-HRMS *m/z* 471.3471 [M-H]^−^ (calcd. for C_30_H_47_O_4_, 471.3474).

### 2.4. Parasites

*L. (L.) infantum*-synonymy *L. (L.) chagasi* (MHOM/BR/72/46) was kindly provided by Prof. Dr. Fernando Tobias Silveira, from the cryobank of the Leishmaniasis Laboratory Prof. Dr. Ralph Laison, Department of Parasitology, Ministry of Health, *Instituto Evandro Chagas* (Belem, Para-Brazil). They were identified using monoclonal antibodies and isoenzyme electrophoretic profiles at the Leishmaniasis Laboratory of the *Instituto Evandro Chagas*. Stationary phase promastigotes were used throughout the entire study. Parasites were maintained in Schneider's Medium (Sigma Aldrich, Germany), supplemented with 10% heat-inactivated fetal bovine serum and 50,000 IU/mL penicillin, 50 *μ*g/mL streptomycin (S10). Promastigote forms in the stationary phase of growth were used in all experiments.

### 2.5. Promastigote and Cytotoxic Assays

Promastigote forms of *L. (L.) infantum* (2 x 10^6^ promastigotes/well) were incubated in a 96-well culture plate in S10 with maslinic acid and miltefosine (0.781 to 100 *μ*g/mL) or amphotericin B (0.016 to 1 *μ*g/mL). Control group was cultivated in medium and vehicle solution (PBS plus 1% DMSO). The parasites were incubated for 24 h at 25°C, and then washed with 200 *μ*L of PBS three times with centrifugation at 3000 rpm, 10 min at 4°C, followed by addition of MTT (3-(4, 5-dimethylthiazol-2-yl)-2,5-diphenyltetrazolium bromide) (10 mg/mL). Four hours later, 50 *μ*L of 10% sodium dodecyl sulfate (SDS) was added to each well. The plates were further incubated for 18 h and read in an ELISA reader at 595 nm. Effective concentration 50% (EC_50_) was estimated using the nonlinear regression test with GraphPad Prism 5.0 software.

Peritoneal macrophages from golden hamsters (*Mesocricetus auratus*) at 10^6^ macrophages/well were cultured in 96-well plates in RPMI 1640 medium supplemented with 10% of fetal bovine serum (Thermo Fisher, USA), 2 mM L-glutamine (Sigma-Aldrich, USA), 10 mM Hepes (Sigma-Aldrich, USA), 1 mM sodium pyruvate, 1% v/v nonessential amino acid solution (Thermo Fisher, USA), 10 *μ*g/mL of gentamicin (Thermo Fisher, USA), and 1000 U/mL of penicillin (Thermo Fisher, USA) (R10 medium). Maslinic acid, miltefosine, or amphotericin B (0.781 to 100 *μ*g/mL) was added and macrophages were incubated at 37°C, 5% CO_2_, during 24 h. Cells were centrifuged at 1000 rpm for 10 min at 4°C and washed 3 times; then cell viability was assessed by the oxidation of MTT. Four hours later, 50 *μ*L of 10% sodium dodecyl sulfate (SDS) was added to each well. The plates were further incubated for 18 h and read in an ELISA reader at 595 nm. Cytotoxic concentration 50% (CC50) was estimated using the nonlinear regression test with GraphPad Prism 5.0 software. The index of selectivity (SI) was estimated according to Yamamoto and collaborators [16]. This study was carried out in strict accordance with the recommendations of the guide for Care and Use of Laboratory Animals of the Brazilian National Council of Animal Experimentation (http://www.cobea.org.br). The protocol was approved by the Ethics Committee of Animal Experiments of the Institutional Committee of Animal Care and Use at the Medical School of São Paulo University (056/16).

### 2.6. Activity of Maslinic Acid in Intracellular Amastigotes, Nitric Oxide, and Hydrogen Peroxide Production

Peritoneal macrophages from golden hamsters (10^6^ macrophages) were collected and cultured on round coverslips in 24-well plates for 4 h in R10 medium. After some washes with warm PBS, cells were infected with *L. (L.) infantum* promastigotes (10 parasites per peritoneal macrophage). The plates were incubated overnight at 5% CO_2_ at 37°C. Maslinic acid and miltefosine were added to the infected macrophages at 7.5, 15, and 30 *μ*g/mL and amphotericin B at 0.01, 0.1, and 1 *μ*g/mL. Twenty-four hours later, macrophage supernatants were collected and stored at −80°C for quantification of nitric oxide (NO) (Life Technologies, USA) and hydrogen peroxide (H_2_O_2_) (Life Technologies, USA) according to the manufacturer's instructions. The coverslips were dried at room temperature, fixed in MeOH, and stained by Giemsa. The number of infected macrophages and parasites per macrophage was determined at least in 100 cells. The infection index (II) was expressed as the percentage of infected macrophages multiplied by the average number of amastigotes per macrophage according to Passero and collaborators [24]. As a positive control for NO and H_2_O_2_, macrophages were incubated with LPS (100 ng/mL) according to [25].

### 2.7. Ultrastructural Alterations Induced by Maslinic Acid in *L. (L.) infantum*

Promastigote forms of *L. (L.) infantum* (2 x 10^6^ promastigotes/well) were incubated in a 24-well culture plate in S10 with maslinic acid, miltefosine, or amphotericin B at the respective EC_50_ values. The control group was cultivated with medium and vehicle solution. Parasites were centrifuged at 3000 rpm, 4°C, 10 min, and washed three times with 200 *μ*L of PBS. Then the pellets were resuspended in glutaraldehyde 2% in 0.1 M sodium cacodylate buffer (pH7.4) and incubated at 4°C, for 60 min. Parasites were postfixed in 1% osmium tetroxide. The samples were washed, followed by dehydration in a graded series of acetone. Then, the samples were embedded in a polyester resin, thin-sectioned in a Reichert ultramicrotome, and double-stained with 7% uranyl acetate and lead citrate aqueous solution (Ladd Research Industries). Stained parasites were examined with a JEOL 1010 (Tokyo, Japan) transmission electron microscope (TEM).

### 2.8. Cell Membrane Damage and Mitochondrial Membrane Potential Assays

Promastigote forms of *L. (L.) infantum* (2 x 10^6^ promastigotes/well) were incubated in 96-well black culture plates (Corning Inc, USA) in S10 at EC_50_ of maslinic acid, miltefosine, or amphotericin B for 24 h, followed by the addition of Sytox Green at 0.5 *μ*M/well (Life Technologies, USA) or Rhodamine 123 at 3 *μ*M/well (Sigma-Aldrich, USA) to evaluate cell membrane damage or mitochondrial membrane potential, respectively. Parasites were placed in the dark for 15 minutes at 25°C, centrifuged at 1200 g, 5 min at 10°C, and washed three times with 200 *μ*L of PBS. Plates containing parasites stained with Sytox Green were read in a fluorescence reader using 530 nm emission wavelength and 490 nm excitation, and parasites stained with Rhodamine 123 read with 520 nm emission and 485 nm excitation wavelengths. Results were normalized concerning the respective control (non-treated parasites). Triton X-100 at 0.05 *μ*L (Sigma Aldrich, USA) was used as a positive control for cell membrane damage and oligomycin at 0.1 *μ*M/well (Cayman Chemicals, USA) as a positive control of mitochondrial membrane potential inhibition.

### 2.9. Statistical Analysis

Data were reported as the mean of three independent assays. Values are expressed as means ± standard deviation. Statistical analyses were performed using GraphPad Prism 5.0 software, and the nonparametric Mann–Whitney test was used to assess the differences between the treated groups and the control group (untreated). Statistical significance was set at a *p* value <0.05.

## 3. Results

### 3.1. Chemical Characterization of Maslinic Acid

**Figure 1:**
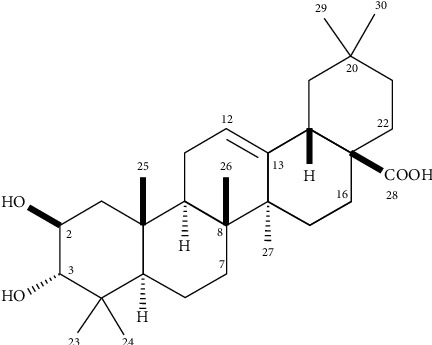
Chemical structure of maslinic acid, isolated from the MeOH extract from the leaves of *H. canum.*

ESI-HRMS spectrum of the isolated compound displayed the [M–H]^−^ ion peak at *m/z* 471.3471, corresponding to the molecular formula C_30_H_48_O_4_, suggesting the occurrence of a triterpene derivative. ^1^H NMR spectrum displayed, besides other overlapped signals at range *δ* 1.07–2.05, seven singlets of methyl groups at *δ* 1.15 (H-27), 1.03 (H-23), 1.00 (H-25), 0.93 (H-30), 0.91 (H-29), 0.84 (H-24), and 0.78 (H-26) as well as two signals attributed to oximethine hydrogens at *δ* 3.71 (m, H-2) and 3.01 (d, *J* = 9.5 Hz, H-3). These data, in association with a triplet at *δ* 5.32 (*J* = 3.7 Hz, H-12), indicated an oleanane triterpene derivative [26]. ^13^C NMR spectrum exhibited 30 signals attributed to seven methyl, nine methylene, six methine, two oxygenated being at *δ* 83.4 (C-3) and 68.5 (C-2) as well as one sp^2^ at *δ* 122.0 (C-12), and eight quaternary carbons, two sp^2^ being at *δ* 144.7 (C-13) and 178.5 (C-28), this last characteristic of carboxylic acid [27]. The comparison of the obtained data with those reported in the literature [28] allowed the identification of maslinic acid (Figure 1).

### 3.2. Antileishmanial and Cytotoxic Activities of Maslinic Acid

**Figure 2:**
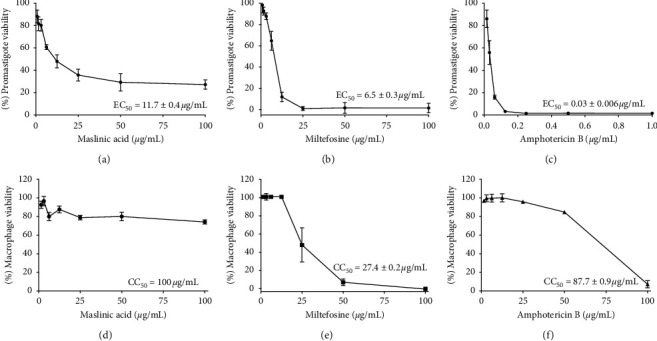
Leishmanicidal and cytotoxic activity. Promastigote forms of *L. (L.) infantum* were incubated with different concentrations of maslinic acid (a), miltefosine (b), or amphotericin B (c) for 24 h; in each of these treatments, the effective concentrations 50% (EC_50_) were estimated. In addition, peritoneal macrophages from golden hamsters were also incubated with maslinic acid (d), miltefosine (e), or amphotericin B (f) for 24 h, and the cytotoxic concentration 50% (CC_50_) was calculated. These experiments were repeated three times, and the values are represented as mean ± standard deviation.

Maslinic acid, miltefosine, and amphotericin B were active against promastigote forms of *L. (L.) infantum*, presenting dose-dependent antileishmanial activities (Figures 2(a)–2(c), respectively). Comparatively, maslinic acid showed the lowest activity on promastigote forms (EC_50_ = 11.7 ± 0.4 *μ*g/mL), in comparison with miltefosine (EC_50_ = 6.5 ± 0.3 *μ*g/mL) and amphotericin B, that was highly active on promastigote forms (EC_50_ = 0.030 ± 0.006 *μ*g/mL).

Maslinic acid was not toxic to peritoneal macrophages from golden hamsters (Figure 2(d)), by contrast, miltefosine, and amphotericin B killed macrophages in a dose-dependent manner, and in this case miltefosine displayed the highest cytotoxic potential (CC_50_ = 27.4 ± 0.2 *μ*g/mL), followed by amphotericin B (CC_50_ = 87.7 ± 0.9 *μ*g/mL), as shown in Figures 2(e) and 2(f), respectively.

Based on these data, it was possible to estimate that against promastigote forms of *L. (L.) infantum*; amphotericin B displayed the highest selective index (SI = 2923.3), followed by maslinic acid (SI = 8.6) and miltefosine (SI = 4.2).

**Figure 3:**
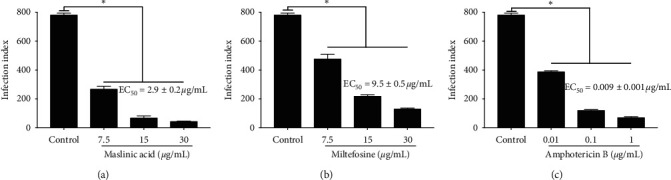
Anti-amastigote activity of maslinic acid. Peritoneal macrophages from golden hamsters were isolated and infected with promastigote forms of *L. (L.) infantum* at 10 : 1 ratio. Twenty-four hours later, infected macrophages were treated with different concentrations of maslinic acid (a), miltefosine (b), and amphotericin B (c). Infected cells were incubated with the drugs for 24 h and their infection indexes were estimated, as well as the effective concentration 50% for each treatment. *∗p* < 0.05 indicates a significant difference concerning the control group.

Maslinic acid (Figure 3(a)) as well as miltefosine (Figure 3(b)) and amphotericin B (Figure 3(c)) exhibited anti-amastigote activity in a dose-dependent manner. Comparatively, maslinic acid presented intermediated antileishmanial activity (EC_50_ = 2.9 ± 0.2 *μ*g/mL), being more active than miltefosine (EC_50_ = 9.5 ± 0.5 *μ*g/mL) and less active than amphotericin B (EC_50_ = 0.009 ± 0.001 *μ*g/mL).

Considering data on the anti-amastigote action of these compounds, the estimated selective indexes for maslinic acid, miltefosine, and amphotericin were 34.5, 2.9, and 9744.4, respectively.

Infected macrophages treated with maslinic acid, miltefosine, or amphotericin B did not produce quantifiable levels of nitric oxide or hydrogen peroxide.

### 3.3. Ultrastructural Changes in Promastigote Forms Treated with Maslinic Acid

**Figure 4:**
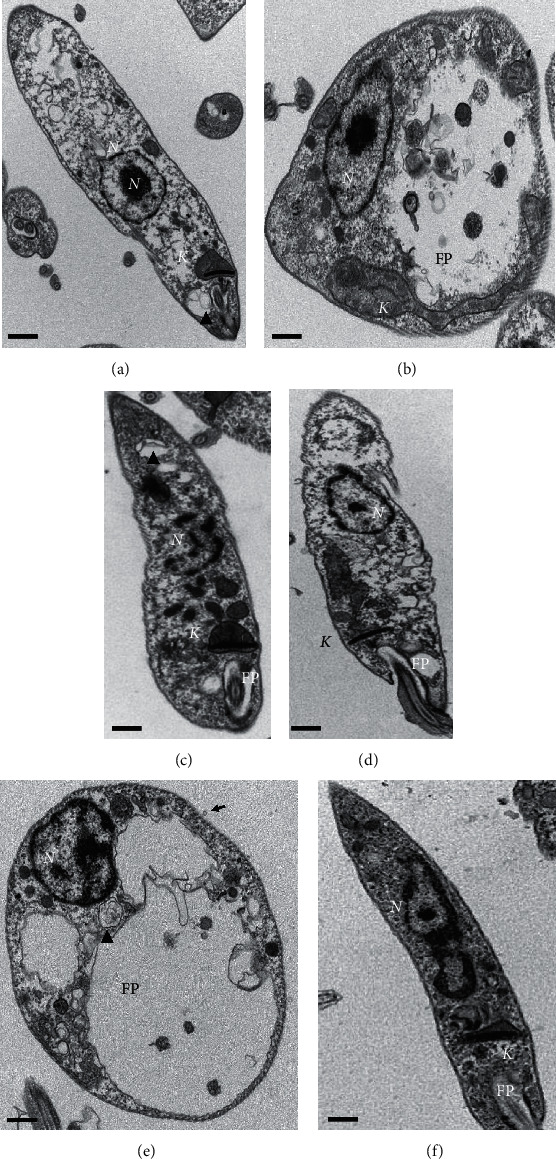
Ultrastructural changes induced by maslinic acid a b, and c, miltefosine d, or amphotericin B e in promastigote forms of *L. (L.) infantum.* Promastigote forms were incubated with EC_50_ of maslinic acid A, B, and C, miltefosine D, or amphotericin B E for 24h; then morphological changes were recorded. Control parasites are shown in F. N, nucleus; FP, flagellar pocket; K, kinetoplast. Black arrow indicates blebs in the cell membrane. Black arrow head indicates compartmentalized membrane debris.

Promastigote forms treated with maslinic acid are shown in Figures 4(a)–4(c). Parasites did not display important changes in the cell membrane (Figure 4(a)), but the cytoplasm presented signs of degeneration with areas containing membrane debris (Figures 4(a) and 4(b)-black arrowhead) associated with an intense exocytic activity in the flagellar pocket (FP) zone (Figure 4(c)). In the complex kinetoplast- (K-) mitochondria (M), it was verified blebs in kDNA (Figure 4(b)) and disruption of mitochondrial cristae (Figure 4(c)); however, some parasites showed preserved morphology of the kDNA and mitochondria (Figure 4(a)). The nucleus of promastigote forms was affected by maslinic acid and the chromatin seems to be fragmented (Figure 4(a)) and in some specimens, the nucleus was completely degraded (Figure 4(c)).

The morphology of *L. (L.) infantum* was affected by miltefosine. The cytoplasm of the parasites was degraded, and blebbing was detected in the area of kDNA (K) (Figure 4(d)), mitochondrial cristae were disorganized (Figure 4(d)). *L. (L.) infantum* treated with amphotericin B lost the fusiform morphology, small blebs were detected attached to the cell membrane (black arrow), and the cytoplasm seemed to be degraded, presenting areas of cytoplasm extraction. Membrane debris (arrowhead) was detected, suggesting that autophagic vacuoles were triggered during the treatment with amphotericin B. Membrane debris was also detected in the flagellar pocket (FP), suggesting intense phagocytic activity (Figure 4(e)).

Control promastigote forms showed a preserved external structure, with a fusiform shape and intact cell membrane (Figure 4(f)). The cytoplasm, flagellum, flagellar pocket (FP), kinetoplast (K), and the nucleus (N) showed regular morphology (Figure 4(f)).

### 3.4. Cell Membrane Integrity and Mitochondrial Membrane Potential Assays

**Figure 5:**
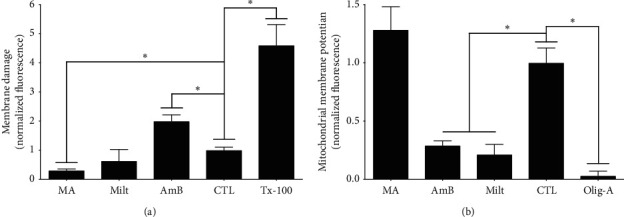
Cell membrane integrity (a) and mitochondrial membrane potential assays (b). Promastigote forms of *L. (L.) infantum* were treated with EC_50_s of maslinic acid, miltefosine, and amphotericin for 24 h; then cell membrane integrity A and mitochondrial membrane potential were analyzed using the probes Sytox green and rhodamine 123, respectively. Triton X-100 was used as a positive control of membrane damage, and oligomycin A as an inhibitor of the mitochondrial membrane potential. ^*∗*^*p* < 0.05 indicates a significant difference.

Parasites treated with maslinic acid showed reduced fluorescence in comparison with the control group (*p* < 0.05), but parasites treated with amphotericin B displayed higher fluorescence intensity compared to the control (*p* < 0.05), as shown in Figure 5(a), suggesting that amphotericin B altered the integrity of the parasite cell membrane. On the other hand, this effect was not observed in parasites incubated with miltefosine.

Maslinic acid did not alter mitochondrial membrane potential in *L. (L.) infantum* promastigotes; in contrast, amphotericin B and miltefosine significantly (*p* < 0.05) inhibited mitochondrial membrane potential in comparison with the control (Figure 5(b)).

## 4. Discussion

In the present work, maslinic acid was isolated from the leaves of *H. canum*, a plant used to treat different medical conditions in traditional communities in Brazil. This compound was chemically identified by NMR analysis and ESI-HRMS data followed by comparison of the obtained data with those described in the literature [28]. Although several species of *Hyptidendron* accumulate bioactive molecules, currently, few works investigated the pharmacological activity of this genus, and despite the microbicide effect reported by the traditional populations, there are not available studies showing the leishmanicidal activity of bioactive molecules from *H. canum*.

Maslinic acid was active on promastigote forms of *L. (L.) infantum*, but it was less active than miltefosine and amphotericin B, both used in the treatment of leishmaniasis. Although both miltefosine and amphotericin have had higher leishmanicidal activity than maslinic acid on promastigote forms, it was possible to observe that miltefosine and amphotericin B displayed high and moderate cytotoxic activity on peritoneal macrophages, respectively. In contrast, cytotoxic events were not observed when peritoneal macrophages from golden hamsters were incubated with maslinic acid, and it positioned this triterpene as active and selective toward promastigote forms. A previous study reported that maslinic acid, isolated from the leaves of olive tree [29], displayed activity against an European strain of promastigote forms of *L. (L.) infantum* with EC_50_ values of 9.32 ± 1.65 *μ*g/mL when parasites were incubated in the interval of 48–72 h; comparatively, maslinic acid eliminated the Brazilian strain of *L. (L.) infantum* at 24 h with EC_50_ of 11.7 ± 0.4 *μ*g/mL. These data indicate that this triterpene has multispecies activity and the Brazilian strain of *L. (L.) infantum* may be more susceptible than the European species. Although maslinic acid exhibited absent or reduced cytotoxic activity on peritoneal macrophages from golden hamsters, an experimental model able to mimic natural infection [18], a previous study showed that this triterpene had a significant cytotoxic activity towards J774 macrophage [30], that is a tumor cell lineage, and thus the differences in the cytotoxicity data may be associated with the cell type employed. Taken together, our data suggest that maslinic acid could be considered an important molecule to develop new prototypes for the treatment of leishmaniasis.

Maslinic acid also impacted the survival of intracellular amastigote forms, and the EC_50_ (2.9 ± 0.2 *μ*g/mL) of this triterpene was 3.3 times higher at eliminating amastigote forms than miltefosine. In the study conducted by Sifaoui and collaborators, maslinic acid also displayed an EC_50_ of 2.90 ± 0.06 *μ*g/mL on intracellular forms of *L. (L.) infantum* isolated in the European continent, and although the EC_50_ value was the same as that we found herein, the method to analyze the activity of maslinic acid was different, since herein we analyzed the infection index, that considers the number and morphology of intracellular amastigote forms, while Sifaoui and collaborators used the Alamar blue method. In this method, amastigotes are differentiated into promastigote forms at 25°C, and then the reagent Alamar blue is used to quantify promastigote viability. However, this reagent analyzes the activity of mitochondria rather than parasite viability. Although interesting, this methodology may not be appropriate to analyze the activity of drugs on intracellular amastigotes. Thus, based on the number and mitochondria activity, the potency of maslinic acid was determined. The infection index is the most used method to analyze the activity of drugs on amastigote forms, and although Alamar blue is an innovative technique to analyze anti-amastigote activity of drugs it is not widely accepted. Taking into consideration that maslinic acid showed superior anti-amastigote activity compared to miltefosine and in the present work it showed a selective index of 34.5, it becomes possible to emphasize that this triterpene is an interesting molecule to develop a new drug to treat visceral leishmaniasis in both new and old worlds.

In addition, it was observed that maslinic acid showed a higher selective index (SI = 34.5) over intracellular amastigote forms than miltefosine (SI = 4.2); however amphotericin B still displayed the highest SI on amastigote forms. Despite the highest activity and selectivity of amphotericin B on amastigotes, a mild toxicity of this drug was observed on macrophages from golden hamsters; additionally experimental [17, 31] and clinical [32, 33] studies already demonstrated severe side effects induced by amphotericin B during the treatment, that in turn have been associated with the low adherence to the treatment. In contrast, maslinic acid is present in different edible vegetables, commonly used in the human diet; thus it does not seem toxic for humans [20, 22, 34]. Furthermore, it was demonstrated that daily oral doses of maslinic acid did not alter the body weight, biochemical, or hematological parameters of experimental animals [35], and in diabetic rats this triterpene exhibited hepatic and renal protective properties [36, 37], suggesting that this triterpene is safe to be used in different medical conditions, including leishmaniasis.

Ultrastructural changes observed in promastigote forms of *L. (L.) infantum* incubated with maslinic acid and miltefosine were related to cytoplasm degradation and disruption of intracellular organelles, especially the mitochondria, which showed an increased volume, associated with disorganization of cristae. Furthermore, blebs were detected in the kDNA-mitochondria complex, suggesting that maslinic acid may target the mitochondria. In addition, the nucleus seems to be affected by this triterpene, since chromatin fragmentation has been detected, and in some individuals the nucleus seems to be completely fragmented, as observed in Figure 4(c), suggesting that maslinic acid also may affect the nucleus of *L. (L.) infantum* promastigote forms. In addition to these morphological changes, membrane debris was observed in the flagellar pocket of parasites treated with maslinic acid. Possibly, similar structural changes observed in parasites treated with maslinic acid or miltefosine may be associated with a correlated mechanism of death.

It was demonstrated that *Leishmania* sp. treated with miltefosine displayed morphological changes compatible with programmed cell death [38, 39], and biochemical changes attested that the parasites underwent apoptosis upon treatment [40–42]. Similarly, a chemically related triterpene, ursolic acid, also induced morphological and molecular changes in treated parasites, resembling apoptosis [7, 16]. Thus, considering the structural similarity between both triterpenes and the similar morphological changes observed in parasites after treatment, it may be possible that maslinic acid triggers programmed cell death in *L. (L.) infantum*. Previously, it was demonstrated that maslinic acid may induce apoptosis in *L. (L.) infantum*, since parasites incubated with IC_90_ exposed phosphatidyl serine on the cell membrane, decreased the mitochondrial membrane potential as well as ATP levels [29]; however, no morphological evidence of programmed cell death was previously demonstrated. In addition, the apoptotic process triggered by maslinic acid was identified in tumor cell strains that displayed plasma membrane disintegration and nuclear fragmentation [43]. In contrast, it has been observed that the leishmanicidal activity of amphotericin B is related to the interaction of the drug with the cell membrane of parasites, causing changes in the integrity of the cell membrane [44, 45]. The main change in the cell membrane of parasites treated with amphotericin B was associated with the presence of small blebs; however pores in the membrane were not observed. In addition, it was verified areas with cytoplasm extraction and autophagic vacuoles, suggesting that amphotericin B degraded lipids [46, 47].

Despite the intracellular disorganization, maslinic acid did not change the integrity of the cell membrane of promastigote forms, as evaluated by morphology and SYTOX Green probe. By contrast, it was observed a significant decrease in the fluorescence units in parasites incubated with maslinic acid than in control. A possible explanation may be related to the morphology of the parasite nucleus. Upon treatment with maslinic acid (at EC_50_ value), it was observed that the nucleus was altered and, in some individuals, it was completely fragmented, that may have affected the potential of SYTOX green probe to bind to the nucleus and emit fluorescence. In contrast, Sifaoui and collaborators [29] observed that maslinic acid was able to damage the cell membrane of *L. (L.) infantum promastigotes*; however the highest time point recorded was 100 minutes, suggesting that cell damage is an early event triggered by maslinic acid. Unlike maslinic acid, the parasites treated with amphotericin B and incubated with Sytox displayed high levels of fluorescence, suggesting that this drug altered the integrity of the cell membrane, allowing the probe to access the nucleus of the parasites, which in this specific situation was more preserved than the nucleus of parasites treated with maslinic acid.

In this study, amphotericin B and miltefosine were able to inhibit the mitochondrial membrane potential, which is crucial for the generation of ATP in the respiratory chain, and as a consequence cells become depleted of energy with subsequent death [29, 48]. In contrast, maslinic acid did not change mitochondrial membrane potential, that was not expected, because it was observed that promastigote forms displayed morphological changes in the mitochondria. This can be explained in some ways: firstly, the morphological changes induced by maslinic acid at the EC_50_ were not enough to alter the mitochondrial membrane potential; secondly, the morphological changes in the mitochondria were observed only in some parasites, and thus the normal parameters observed could be associated with the parasites presenting preserved mitochondria; third, maslinic acid may not target mitochondria at the EC_50_, and the morphological changes observed in the mitochondria of some parasites may be associated with a late process of death, in which all organelles are affected.

Taken together, *in vitro* data demonstrate that maslinic acid was able to eliminate promastigote forms and it exerted an important activity in the elimination of amastigote forms of *L. (L.) infantum* in a selective manner, with an action superior to that of miltefosine. Additionally, morphological data suggest that this triterpene would be acting by inducing programmed cell death in *L. (L.) infantum*. These results highlight that maslinic acid may be an interesting therapeutic alternative in the treatment of leishmaniasis.

## Data Availability

The data used to support the findings of this study are available from the corresponding author upon request.
